# The AI recommendation paradox: a systematic review evaluating the promise, peril, and path forward for large language models in exercise recommendation

**DOI:** 10.5114/biolsport.2026.158676

**Published:** 2026-03-04

**Authors:** Tianyuan He, Di Lu, Yongye Ma, Jiaxin He, Duanying Li, Guoxing Li, Jian Sun

**Affiliations:** 1 Guangzhou Sport University, Guangzhou, Guangdong, China; 2 Key Laboratory of Human-Computer Intelligent Interaction for Athletic Performance and Health Promotion, China

**Keywords:** Exercise prescription, Safety, Efficacy, Human-computer interaction, LLM

## Abstract

Large Language Models (LLMs) are rapidly emerging as tools for generating personalized exercise advice, creating an “AI Prescription Paradox” of promising potential but significant risks. This study systematically reviews the empirical evidence to evaluate the efficacy, quality, and safety of LLMs in exercise prescription. Following PRISMA guidelines, we conducted a systematic review of 24 empirical studies (N = 2,512 participants) published up to June 19, 2025. Data were extracted from human intervention trials, in silico expert evaluations, and human-computer interaction studies, and a comprehensive narrative synthesis was performed. Our synthesis reveals significant deficits. In head-to-head trials comparing AI to human experts, LLM-generated plans were inferior in 5 out of 6 (83%) cases for driving physiological adaptations. Most critically, systemic safety flaws were identified in 14 of 24 studies (58%), with models recommending contraindicated exercises for clinical populations. While the quality of AI advice was highly variable, novel conversational and context-aware models showed promise for user engagement. LLMs in their current state are powerful assistive tools but cannot safely replace the core decision-making and supervisory roles of human experts. We advocate for a shift towards a human-AI synergistic paradigm. To guide this, we propose a novel, evidence-based risk stratification framework to help practitioners harness these tools safely and effectively, ensuring that professional oversight remains paramount.

## INTRODUCTION

Regular physical activity is a cornerstone for maintaining and promoting physical and mental health and for preventing and managing numerous non-communicable diseases (e.g., cardiovascular disease, type 2 diabetes, certain cancers, and mental health disorders), a consensus widely advocated by the global health community [1, 2]. Personalized exercise prescription, defined as a guidance plan tailored to an individual’s health status, fitness level, preferences, goals, and life context, is considered a key strategy to maximize benefits, enhance adherence, and ensure safety [3]. However, despite its undisputed value, access to high-quality, personalized exercise guidance from qualified professionals (e.g., physicians, physical therapists, certified personal trainers) remains challenging for the public, particularly for those in resource-limited settings or facing economic constraints [4].

In recent years, the rapid advancement of artificial intelligence technologies, epitomized by Large Language Models (LLMs) (e.g., those based on the seminal Transformer architecture [5]) for which GPT is a notable example, has presented an unprecedented opportunity to address these challenges. Leveraging their powerful capabilities in natural language understanding, information synthesis, content generation, and fluid user conversation, LLMs are rapidly permeating various sectors, including health care [6]. In the exercise and health domain, their potential is particularly compelling: in theory, they could rapidly process vast amounts of exercise science knowledge, clinical guidelines, and individual user data to generate preliminary personalized exercise plans and recommendations. They could even interact with users as chatbots or virtual coaches, providing immediate feedback and motivational support. This technological prospect appears to herald the possibility of “democratizing” access to professional exercise prescription in a low-cost, highly efficient manner [7].

Behind this promising outlook, however, initial exploratory studies and early applications have begun to expose the potential perils and complexities of using LLMs for personalized exercise recommendation. Some research indicates that plans directly generated by general-purpose LLMs might have deficiencies in scientific validity, accuracy, and completeness [8], and may even provide inappropriate or potentially unsafe recommendations, especially when dealing with individuals with complex health conditions or special needs [9]. For instance, LLMs may struggle to accurately assess an individual’s exercise risk, identify contraindications, or dynamically adjust training loads based on subtle physiological feedback. Furthermore, the readability of LLM outputs can far exceed the health literacy levels of the general public [10], the relationship between user trust and actual adoption of advice remains unclear [11], and the methods for sustaining user engagement and adherence over the long term are pressing human-computer interaction (HCI) questions that require in-depth investigation.

While the field is rapidly evolving, recent research has largely focused on isolated evaluations or broader healthcare applications. Notable empirical studies, such as those by Düking et al. [12] and Havers et al. [13], have specifically evaluated the quality and reproducibility of running and resistance training plans generated by LLMs like ChatGPT. These studies provide critical insights—particularly regarding the models’ dependence on detailed prompting—but they remain single-context evaluations. Broader syntheses published between 2022 and 2025 have begun to map the landscape [14–17], yet they often focus on general medical chatbots or traditional machine learning algorithms rather than the specific capabilities of generative AI in exercise prescription. To date, no study has systematically integrated findings across diverse methodological domains—including human interventions, *in silico* expert evaluations, and HCI studies—to provide a holistic view of this technology.

Consequently, the specific evidence base for LLMs in personalized exercise recommendation remains fragmented. This systematic review aims to fill this gap by comprehensively synthesizing the available empirical research. Unlike previous works, we not only evaluate efficacy and quality but also operationalize a novel, clinicallyoriented risk stratification framework to guide the safe implementation of these tools. The specific objectives of this study are:

1. To systematically review and summaries the current evidence on the physiological efficacy and performance-enhancing effects of LLM-generated personalized exercise recommendations.
2. To critically evaluate the content quality of LLM-generated exercise recommendations, including their alignment with established guidelines, accuracy, comprehensiveness, readability, and the influence of different LLM models and prompting strategies on output quality.
3. To investigate the safety of LLM-generated exercise recommendations, including their ability to identify contraindications, the appropriateness of their advice for special populations (e.g., those with comorbidities), and the risk of providing potentially harmful suggestions.
4. To explore the user-centered factors (e.g., trust, acceptance, user experience) and the key methodological and technical factors (e.g., interaction modalities, data integration, risk mitigation strategies) that influence the effectiveness of LLM applications.

Through a systematic and critical analysis of these questions, this review aims to delineate the current capability boundaries of LLMs in this field, identify critical knowledge gaps and research challenges, and provide valuable, evidence-based recommendations for future research, technological development, clinical practice, and policymaking. More importantly, this review seeks to identify critical “early warning signals,” to provide a prudent starting point and an actionable framework for responsible innovation and safe application in this domain, thereby ensuring that the potential of AI is translated into safe and effective health benefits.

## MATERIALS AND METHODS

### Study Design and Registration

This systematic review was conducted and reported in accordance with the Preferred Reporting Items for Systematic Reviews and Meta-Analyses (PRISMA) 2020 statement [18]. The review protocol was prospectively registered with the International Prospective Register of Systematic Reviews (PROSPERO) on June 18, 2025 (registration number: CRD420251075636).

Recognizing that the term “prescription” carries legally regulated medical connotations in many jurisdictions, this review systematically uses the more neutral terms “exercise recommendation” or “training plan” when describing LLM-generated content. The term “prescription” is used only when citing original sources or referring specifically to plans issued by legally qualified professionals.

### Eligibility Criteria

The eligibility criteria were defined using the PICOS (Population/ Problem, Intervention, Comparator, Outcome, Study Design) framework. We broadly included empirical studies that used an LLM to generate exercise recommendations for human participants (healthy or clinical) or for *in silico* personas, excluding studies focused solely on acute therapeutic rehabilitation. The intervention was any LLMbased system for exercise guidance. Comparators were diverse, including human experts, guidelines, or no comparator. Primary outcomes were efficacy, quality, and safety; secondary outcomes included user-centered and technical metrics. A wide range of study designs, from RCTs to qualitative analyses, were eligible. Detailed inclusion and exclusion criteria for each PICOS element are provided in Appendix B.

### Information Sources and Search Strategy

A systematic search was conducted up to June 19, 2025, in major electronic databases including PubMed, Scopus, Web of Science, and IEEE Xplore, alongside comprehensive searches for grey literature. The search strategy was constructed around four core concepts: (1) AI technologies, (2) the exercise domain, (3) the application process, and (4) evaluation outcomes. The full, unabridged search strategy for each database, including all Boolean operators and keywords, is available in Appendix A.

### Study Selection

**FIG. 1:**
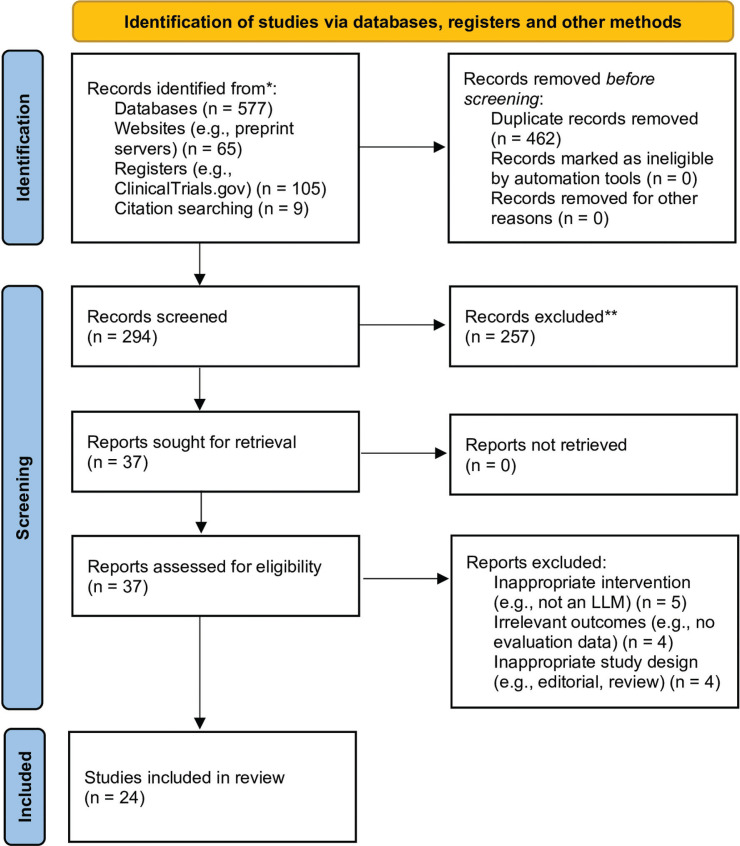
PRISMA 2020 flow diagram for the systematic review. The diagram illustrates the flow of information through the different phases of the review. It maps out the number of records identified from various sources (databases, registries, citation searching), the number of records included and excluded at each stage of screening, and the specific reasons for exclusions during the full-text eligibility assessment phase.

All retrieved records were imported into Zotero for deduplication. Subsequently, two independent reviewers (Author 1, Author 2) screened the titles and abstracts of all unique records against the predefined eligibility criteria. Full texts of potentially eligible articles were retrieved for a second round of independent assessment by the same two reviewers. Any disagreements during screening and eligibility assessment were resolved through discussion; if consensus could not be reached, a third senior reviewer (Author 3) made the final decision. Inter-reviewer agreement was strong at both the title/ abstract screening stage (Cohen’s κ = 0.88) and full-text eligibility stage (κ = 0.92). The entire study selection process was documented and is presented in a PRISMA 2020 flow diagram (Figure 1) [18].

### Data Extraction

Two reviewers independently extracted data from all 24 included studies using a pre-piloted, standardized data extraction form. Key data points included:

– **Study characteristics:** Authors, year, country, study design, publication venue.
– **Participant characteristics:** Participant type, sample size, age, sex, health status, training level, and the complexity of user personas in *in silico* studies.
– **Intervention details:** The LLM model and version used, specific prompting strategies or interaction modalities (e.g., conversational, RAG), and intervention duration and frequency.
– **Comparator details:** Type of control (human expert, guidelines, other AI, etc.).
– **Outcomes and measurements:** Definitions and methods for measuring primary and secondary outcomes.
– **Safety data:** Any descriptions of adverse events, safety warnings, handling of contraindications, or potentially harmful advice were proactively extracted, regardless of whether they were a primary outcome. For the purpose of this review, ‘safety’ was operationalized based on ACSM guidelines. Recommendations were classified as ‘unsafe’ if they contained absolute contraindications for the specified population, promoted biomechanically hazardous techniques without warning, or failed to include necessary preparticipation screening.
– **HCI and interaction data:** Descriptions of the system interface, interaction flow, and user feedback mechanisms.
– **Key findings and conclusions:** The main findings and conclusions as reported by the authors. Discrepancies in data extraction were resolved by discussion to ensure accuracy and completeness.

### Risk of Bias and Quality Assessment

Two reviewers independently assessed the risk of bias using the Cochrane RoB 2 tool for RCTs and the ROBINS-I tool for non-randomized interventional studies. For other designs, such as *in silico* and qualitative studies, for which standardized tools are lacking, we developed and applied a customized quality assessment checklist. The detailed domains and guiding questions for this custom checklist, based on established principles of methodological rigor and trustworthiness, are provided in Appendix B. Assessment results are summarized in Appendix C.

### Data Synthesis

Given the considerable clinical and methodological heterogeneity across the included studies (in terms of populations, interventions, comparators, and outcomes), a quantitative meta-analysis was deemed inappropriate. Consequently, we employed a narrative synthesis approach, guided by the framework proposed by Popay et al [19]. This process involved: (1) developing an initial theoretical model of LLMs’ role in exercise recommendation (the “Promise and Peril” dualism); (2) conducting a systematic preliminary synthesis and thematic analysis of the extracted data, drawing on principles of Reflexive Thematic Analysis [20], to identify and refine core analytical themes (Efficacy, Quality, Safety, and User-Centered Factors); (3) exploring relationships and heterogeneity within and between studies under these themes; and (4) assessing the robustness of the synthesized findings. The results are presented as descriptive text, supported by summary tables and visualizations, to provide a comprehensive, in-depth, and critical overview of the current evidence.

### Use of AI and AI-assisted technologies

In accordance with journal policy, we declare the use of generative AI in the preparation of this manuscript. Gemini (Google, July 2025 version) was used for the following purposes: (1) to assist in refining the English-language phrasing and ensuring terminological consistency throughout the manuscript after the initial draft was written by the authors; and (2) to help structure the manuscript sections according to the journal’s “Information for Authors” guidelines. For instance, a prompt used was: “Based on the provided ‘Information for Authors’ for The Lancet Digital Health and my draft Methods section, please refine the language to meet the journal’s standards and ensure all required components for a systematic review are clearly articulated.” All AI-generated suggestions were critically reviewed, edited, and validated by the authors, who take full responsibility for the final content of the publication. Crucially, we declare that all data extraction, critical appraisal, and final interpretation of findings were conducted and verified solely by the human authors to ensure accuracy and scientific integrity.

## RESULTS

### Study Selection

The study selection process is detailed in the PRISMA flow diagram (Figure 1). Our initial search identified 756 records. After removing 462 duplicates, 294 records were screened, leading to the retrieval of 37 full-text reports for eligibility assessment. Of these, 13 were excluded, primarily for using a non-LLM intervention, lacking evaluation data, or having an ineligible study design. Ultimately, 24 studies were included in the systematic review.

### Characteristics of Included Studies

**FIG. 2:**
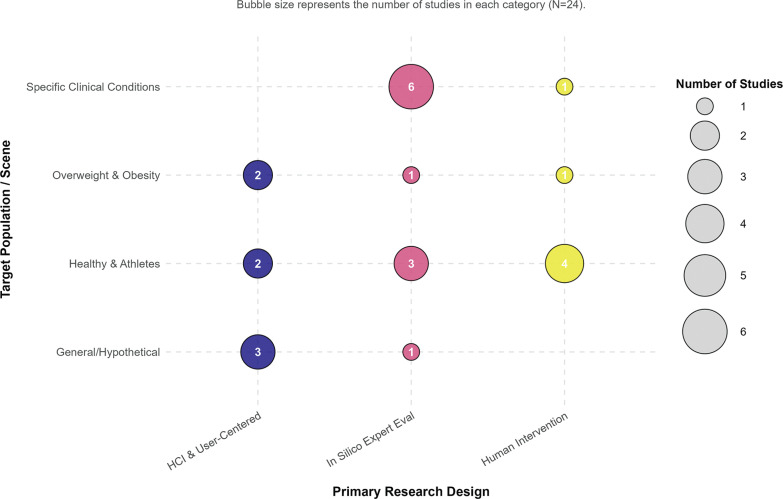
Landscape of Included Studies by Design and Population (N=24). The bubble chart visualizes the evidence base of this systematic review. Each bubble’s size is proportional to the number of studies at the intersection of a specific primary research design (X-axis) and target population or scene (Y-axis). The colors correspond to the three main study design categories: HCI & User-Centered (blue), In Silico Expert Evaluation (pink), and Human Intervention (yellow). The chart highlights key features of the current research landscape, including a concentration of intervention studies in healthy and athletic populations and a predominance of *in silico* studies for assessing scenarios involving specific clinical conditions.

The 24 included studies were geographically diverse and published predominantly between 2023 and 2025. The designs were varied, comprising mainly *in silico* expert evaluations (n = 11), user-centered and HCI studies (n = 7), and human interventions (n = 6). Target populations ranged from healthy athletes to diverse clinical groups, and OpenAI’s GPT series was the most frequently investigated model. Full details of all included studies are presented in Table 1 and visualized in Figure 2.

**TABLE 1:** Characteristics of Included Studies. This table provides a comprehensive overview of the 24 studies included in the systematic review, detailing their origin, design, target population, and key methodological features.

Study ID (Author, Year)	Country/Region(s)	Study Design	Population	Sample Size (N)	LLM(s) Used	Comparator(s)	Key Outcome Focus
Akrimi et al. [27]	Germany	In silico expert eval.	Fictional patients (T2DM + comorbidities)	3 Experts (authors)	ChatGPT-4o	ADA/ACSM guidelines	Quality, Safety

Comendant [26]	Netherlands	Quasi-experimental (pre-post)	Male swimmers (beginner/intermediate)	10 (RAG:5, No-RAG:5)	GPT-4o	LLM without RAG	Efficacy (Skill Acquisition)

Dergaa et al. [9]	Int. Collab.	In silico expert eval. (Quasi-qualitative)	Hypothetical patients (diverse health conditions)	Large international expert panel (authors)	GPT-4	Expert knowledge & guidelines	Quality, Safety

Düking et al. [12]	Germany, et al.	In silico expert eval.	Fictional male runner	10 Endurance coaches	ChatGPT (3.0.1)	Internal (3 AI plans, diff. prompts)	Quality (Prompt Effect)

Ebrahimi et al. [24]	Iran	Quasi-experimental	Blind female students with flatfoot	30 (Exp:15, Control:15)	“Scopus AI” (unclear)	Control group (routine PE)	Efficacy (Special Needs)

Erol & Arıkan [30]	Turkey	In silico expert eval.	General knowledge on core exercises	9 Physiotherapists	ChatGPT 3.5 (Turkish)	N/A (Expert ratings)	Quality (Accuracy)

Genç et al. [22]	Turkey/Kyrgyzstan	RCT	Healthy untrained males	20 (AI:10, PT:10)	GPT-4	Personal Trainer (supervised, dynamic)	Efficacy (Strength)

Haag et al. [34]	Austria, et al.	In silico eval. (Multi-group comparison)	Cardiac rehab patient personas	Generators: 30; Assessors: 38	GPT-4	Laypersons & Healthcare Professionals (HCPs)	Quality (JITAI Generation)

Havers et al.[13]	Germany	In silico expert eval.	Hypothetical advanced male athlete (hypertrophy)	12 Experts	GPT-4, Google Gemini	Internal (LLMs, prompts, reproducibility)	Quality, Reproducibility

Jones et al. [51]	USA	System Design & Pilot Field Study (Qualitative)	Real users with long-term goals	2 (pilot study)	GPT-4 Turbo	Baseline (non-contextual reminders)	HCI (User Experience)

Larbi et al. [52]	Norway/Switzerland	Qualitative (Interviews + Focus Groups)	Adults with morbid obesity	9	N/A (Preferences for future AI)	N/A (Needs analysis)	HCI (User Preferences)

Larbi et al. [53]	Norway/Switzerland	Usability Testing (Think-aloud)	Adults interacting with FysBot app	5 (colleagues)	ChatGPT-3.5 (in FysBot)	N/A (Formative eval.)	HCI (Usability)

Masagca [23]	Philippines	Quasi-experimental	Untrained collegiate students	87 (AI:43, Human:44)	ChatGPT 3.5	Human-made program	Efficacy (Health-Related Fitness)

Oliveira [54]	Portugal	In silico expert eval.	Hypothetical (injury history focus)	16 Experts	ChatGPT-3.5, GPT-4	Personal Trainer (PT) plans	Quality, Safety

Pajo et al. [21]	Philippines	Quasi-experimental	Collegiate volleyball athletes	43 (AI:23, Human:20)	ChatGPT 3.5	Human-made program	Efficacy (Performance)

Philuek et al. [25]	Thailand	RCT (Pilot Study)	Healthy overweight male students	9 (AI:6, Control:3)	ChatGPT 4.0	Control group (no intervention)	Efficacy (Health Metrics)

Saraç et al. [29]	Turkey	In silico expert eval.	Hypothetical obese female	3 Expert trainers	GPT-4, GPT-4o, Gemini-1.5 Pro	Expert trainer program	Quality, Safety

Shin et al. [32]	USA/S.Korea	System Design & Multi-faceted Eval.	Users creating plans; Experts evaluating plans	User study: 18; Expert eval: 3	GPT-4 (in PlanFitting)	N/A (System evaluation)	HCI, Quality

Strömel et al. [33]	Int. Collab.	Online Experiment (RCT)	Fitness tracker users (viewing own data)	273	GPT-4	Data presentation (Chart vs. Text)	HCI (User Reflection)

van Arum et al. [11]	Netherlands	Mixed-methods (Survey + Interviews)	Users in PA decision-making	Survey: 184; Interviews: 24	GPT-4.0 (in MoveAI)	N/A (Exploring human-AI dynamics)	HCI (Trust, Decision-Making)

Wachholz et al. [31]	Austria	Mixed-methods (Survey + Interviews)	Recreational athletes; Beginner runners	Survey: 119; Interviews: 6 coaches	ChatGPT 3.5	Human expert plan	HCI (Trust, Acceptance), Quality

Washif et al. [55]	Int. Collab.	In silico expert eval.	Hypothetical lifters (intermediate/advanced)	N/A (Author eval.)	GPT-3.5, GPT-4	Established guidelines (NSCA)	Quality (Guideline Adherence)

Xu et al. [28]	China	In silico expert eval. (Mixed)	Real patients (hypertension + comorbidities)	24 Multidisciplinary experts	ChatGPT 4.0	Intelligent Health Promotion System (IHPS)	Quality, Safety

Zaleski et al. [8]	USA	In silico expert eval. (Mixed)	26 Clinical populations (ACSM guidelines)	2 Coders	ChatGPT (likely 3.5)	ACSM GETP (gold standard)	Quality (Comprehensiveness, Accuracy)

### Efficacy of LLM-Generated Exercise Interventions

Six human intervention studies assessed efficacy, with findings summarized in Table 2. In head-to-head comparisons, LLM-generated plans were consistently found to be inferior to human-expert programmes for improving athletic performance [21], muscle hypertrophy [22], and cardiovascular fitness [23]. One study also revealed a significant sex disparity, where a plan was ineffective for female participants [23]. However, LLMs showed potential as activation tools against no-intervention controls for specific clinical populations [24, 25] or when enhanced with advanced techniques like Retrieval-Augmented Generation (RAG) for skill acquisition [26].

**TABLE 2:** Efficacy of LLM-Generated Interventions on Physiological and Performance Outcomes. This table summarizes the results from studies that directly tested the efficacy of an LLM-generated exercise program in human participants.

Study ID (Author, Year)	Key Efficacy Metric(s)	Result for AI Group (Summary)	Result for Comparator Group (Summary)	Between-Group Comparison (AI vs. Comparator)	Finding
Genç et al. [22]	Muscle Resistance (Arm/Shoulder); Body Comp. (LBM, Fluid)	Limited. Sig. improvement ONLY in Left Arm Resistance (p < 0.05). No sig. changes in other metrics (p > 0.05).	Effective. Sig. improvements (p < 0.05) in all metrics: Arm/ Shoulder Resistance, Lean Body Mass, Body Fluid.	Human-Expert Group Superior. Human group achieved broad physiological adaptations (strength + hypertrophy) which the AI group failed to achieve.	●↓

Masagca [23]	Flexibility; Cardiovascular & Muscular Endurance	Mixed. Improved Flexibility & Upper-limb endurance (p < 0.05). Crucially, NO sig. improvements in FEMALE participants (p > .05).	Effective. Sig. improvements in Flexibility, Upper & Lower-limb endurance (p < 0.05) for both sexes. Males improved Cardio (p < 0.05).	Human-Expert Group Superior. Human plan led to significantly better Cardiovascular Endurance (p < 0.05) and Male Lower-limb Endurance (p < 0.05) compared to AI.	●↓

Pajo et al. [21]	Vertical Jump (VJ); Horizontal Jump (HJ)	Mixed. Sig. improvement in Right Unilateral VJ (p < 0.01). NO sig. improvement in overall Bilateral VJ (p=0.40) or Bilateral HJ (p=0.07).	Effective. Sig. improvements in overall Bilateral VJ (p=0.02) and Right Unilateral VJ (p=0.04).	Human-Expert Group Superior. Human plan led to significantly higher Bilateral Vertical Jump compared to AI (p=0.05). Human females also outperformed AI females in Left Unilateral HJ (p=0.01).	●↓

Ebrahimi et al. [24]	Balance (Y-Test); Foot Posture (Navicular Drop)	Effective. Sig. improvement in Balance (p=0.035) and reduction in Navicular Drop (p=0.001) in blind female students.	Routine PE (Control): Less effective than the targeted AI program.	AI Superior to Routine Control. AI-generated targeted intervention significantly outperformed standard physical education classes for this special population.	○↑

Philuek et al. [25]	Weight Loss (BMI); Cardiovascular & Functional Fitness	Effective. Sig. improvements in Weight/BMI (p=0.028), Heart Rate Recovery (p=0.026), and Sit-to-stand (p=0.027).	Ineffective (Control). No significant changes in any metrics (p > 0.05).	AI Superior to Control. AI group significantly outperformed the non-intervention control in BMI (p=0.020), Heart Rate Recovery (p=0.019), and Sit-to-stand (p=0.019).	○↑

Comendant [26]	Swimming Technique (Stroke Count); User Experience	RAG-Enhanced AI (LLM-UR): Sig. superior Stroke Count Reduction (p < 0.05), Personalization (p=0.02), and Fun (p < 0.05).	Basic AI (LLM-U): Inferior technical outcome. Sig. higher Motivation (p < 0.05).	RAG Enhancement Superior. Adding Retrieval-Augmented Generation (RAG) significantly improved technical efficiency and personalization compared to the base model.	○↑

Legend:

●↓: AI Inferior (The AI-based intervention showed significantly lower efficacy compared to the human expert comparator).

○↑: AI Effective/Superior (The AI-based intervention showed significant efficacy, outperforming a non-intervention control or a baseline AI model).

Sig.: Statistically Significant (p < 0.05).

LBM: Lean Body Mass.

### Quality and Safety of LLM-Generated Recommendations

Numerous *in silico* studies evaluated the quality and safety of LLM outputs (Table 3). While factual accuracy was often acceptable, a critical and pervasive issue was the poor comprehensiveness of LLM-generated advice [8]. Output quality was highly dependent on prompt detail, yet plans often remained suboptimal even with expertlevel prompts [12, 13]. Readability was another concern, with outputs often requiring a university-level reading ability [8].

**TABLE 3:** Quality and Safety of LLM-Generated Exercise Prescriptions from In Silico and Expert Evaluation Studies. This table details the findings from studies evaluating the quality (e.g., accuracy, adherence to guidelines) and safety of LLM-generated content.

Study ID (Author, Year)	Key Quality Findings (Summary)	Key Safety Findings/Concern(s) (Summary)
Akrimi et al. [27]	Plans met key principles but lacked feasibility and specificity.	**Serious safety issues identified;** recommended contraindicated high-intensity exercise for diabetic retinopathy.

Dergaa et al. [9]	Created “general safety-conscious” plans but lacked precision and effective variability.	**Prioritized “excessive safety over the effectiveness of training,”** potentially rendering plans ineffective.

Düking et al. [12]	Quality was highly dependent on prompt detail, but even the best plan was “sub-optimal.”	The best plan had a progression deemed “too rapid” (injury risk). Health screening rated “neutral.”

Erol & Arıkan [30]	General knowledge on core exercises was good, but poor for individualization.	Scored lowest on identifying **“situations when core exercises are not suitable” (contraindications).**

Haag et al. [34]	**Landmark finding:** GPT-4 JITAIs significantly outperformed both laypersons & HCPs on all quality metrics.	GPT-4 JITAIs were rated highest on professionalism and less likely to evoke negative emotions.

Havers et al. [13]	High reproducibility of plan quality. GPT-4 outperformed Gemini.	Health screening was included in the evaluation criteria.

Oliveira [4]	GPT-4 plans were rated *higher* than PT-developed plans for cases with injury history.	GPT-4 was perceived as superior to human experts for specific injury cases in this evaluation.

Saraç et al. [29]	AI plans were consistent with guidelines but less creative than human expert plans.	**Recommended significantly higher and potentially unsafe initial loads & HR zones** for an obese beginner.

Washif et al. [55]	GPT-4 was more detailed than GPT-3.5, but both required “fine-tuning” by experts.	Plans were “likely” to require modifications before application to ensure safety.

Xu et al. [28]	GPT-4 plans were rated higher than a comparator AI system (IHPS) on most metrics.	GPT-4’s plan involved **“higher health risks”** for a virtual patient with a functional movement disorder.

Zaleski et al. [8]	**Damning findings:** Comprehensiveness was poor (41.2%), and readability required a college level.	The majority of inaccuracies (53%) related to **systematically over-recommending pre-participation medical clearance.**

Systemic safety flaws were a major finding, identified in 14 of the 24 included studies (58%). Studies consistently revealed that LLMs could recommend directly harmful or contraindicated exercises [27, 28], propose unsafe initial training parameters [29], and fail to identify contraindications [30]. A striking example of a contraindicated output was observed in Akrimi et al. [27], where the LLM generated a high-intensity interval training (HIIT) plan for a persona with diabetic retinopathy. The plan included heavy lifting and rapid head movements without warning—activities that dramatically increase intraocular pressure and pose a severe risk of retinal detachment. Similarly, Dergaa et al. [9] reported that models frequently suggested inappropriate exercises, such as ‘heavy deadlifts,’ for users with a history of lumbar disc herniation without advising on necessary modifications. This risk was compounded by an ‘overly-conservative’ paradox, where LLMs would sometimes sacrifice training effectiveness for excessive safety, demonstrating a lack of nuanced clinical judgment [9].

### User-Centered Outcomes and Novel Interaction Paradigms

HCI-focused studies explored user perceptions and novel interaction models. User trust in AI was found to be complex and selective, influenced by prior use and individual decision-making styles [11, 31]. Conversational agents for co-creating plans [32] and LLM-generated narratives for enhancing self-reflection [33] emerged as promising interaction paradigms. Notably, in one large-scale *in silico* evaluation, GPT-4 significantly outperformed both laypeople and healthcare professionals in generating just-in-time adaptive interventions (JITAIs) for a high-risk population, suggesting a unique advantage in dynamic health support tasks [34].

## DISCUSSION

This systematic review of 24 studies provides a clear and sobering depiction of the current reality of large language models (LLMs) in the field of personalized exercise recommendation. The findings systematically reveal significant, and at times fundamental, deficits in the efficacy, quality, and, most critically, safety of LLM-generated outputs. However, this does not imply the technology is without value. On the contrary, a profound understanding of these shortcomings is the logical starting point for abandoning unrealistic narratives of “AI replacement” and instead redefining the role of LLMs to construct a safer and more effective human-AI synergistic paradigm. This discussion will first delve into the root causes of these deficiencies and then argue how LLMs, when correctly positioned as assistive tools under professional supervision, can unlock their unique potential in user empowerment, efficiency enhancement, and innovative interaction design. The structure of this section follows the four core research objectives outlined in the introduction, addressing the limitations in efficacy (Objective 1), the challenges of quality and safety (Objectives 2 & 3), and the user-centered and technical factors influencing their application (Objective 4).

### Summary of Main Findings

This systematic review paints a complex picture of the “promise” and “peril” inherent in this disruptive technology. On one hand, LLMs demonstrate unprecedented potential to enhance access to health information, assist in drafting preliminary exercise recommendations, and enable novel user interaction paradigms, such as conversational guidance and just-in-time adaptive interventions (JITAIs). On the other hand, our synthesis systematically exposes severe deficiencies across several core dimensions. These “perils” manifest as: (1) insufficient evidence for the long-term efficacy of standalone LLM-generated plans in driving physiological adaptations, which are often inferior to those of human experts; (2) significant safety flaws, particularly in handling individuals with complex comorbidities, identifying contraindications, and providing appropriate risk warnings; and (3) a need for substantial improvement in the comprehensiveness, scientific rigor (especially in applying advanced training principles), and user-facing readability of LLM outputs.

Therefore, the central conclusion of this review, based on the available evidence, is that while LLMs hold vast promise as powerful assistive tools, they cannot yet safely or effectively replace the core decision-making and supervisory roles of human exercise and health professionals. This recognition moves us beyond a simple “promise versus peril” dichotomy toward a more constructive framework: a human-AI synergistic paradigm that positions humans at the core, augmented by AI [35, 36]. This forms the central thesis of our review.

Our findings align with observations in other specialized sports domains. For instance, Puce et al. [37] investigated the role of AI digital platforms in optimizing nutrition strategies for ultra-endurance sports. They concluded that while advanced models like ChatGPT-4 demonstrated high accuracy (93%) in answering specialized nutritional queries—often outperforming human groups—their optimal application lies in an ‘integrated approach.’ Specifically, the authors emphasized that AI insights should be combined with expert human judgment to ensure dietary recommendations are not only accurate but also personalized and effective. This reinforces the broader consensus that AI’s optimal role in sports science is one of augmentation, enhancing the capabilities of professionals rather than automating them.

### Efficacy and Limitations of LLMs for Long-Term Physiological Adaptation

A core finding from the limited available intervention research is the apparent efficacy gap between current LLMs and human experts in driving long-term physiological adaptation. We observed a clear pattern: in all head-to-head intervention studies comparing AI-generated plans to human-expert programmes, the AI was found to be inferior, whether for hypertrophy, cardiovascular endurance, or sport-specific performance [21–23]. While some variability in performance can be attributed to the quality of user inputs, consistent with the findings of Havers et al. [13] and Düking et al. [12] which showed that detailed prompts significantly improve output reproducibility and quality, even optimally prompted AI plans were frequently rated as sub-optimal by expert coaches compared to human-designed programs.

This widespread lack of efficacy is not coincidental but points to a deeper, fundamental deficit in the application of core exercise physiology principles by current LLMs. Their generated plans are often static, lacking mechanisms for dynamic adjustment of training volume and intensity based on user feedback, which directly violates the principle of “progressive overload” necessary for long-term adaptation [38]. An advanced human coach employs autoregulation tools like RPE or RIR [39] to ensure optimal stimulus in each session—a capability LLMs currently lack. Furthermore, a deficient understanding of the principles of “specificity” [40] and “individuality”, such as neglecting potential sex-based differences [41] in response to training, further constrains their effectiveness.

Despite these limitations, LLMs show potential in specific contexts, particularly as “activation tools” or “skill-learning aids.” For individuals lacking exercise knowledge or access to professional guidance, “any structured exercise may be better than none.” LLMs can quickly and at low cost provide a foundational framework to help them take the first step [24, 25]. Moreover, when combined with technologies like Retrieval-Augmented Generation (RAG) [42] for skill-learning tasks such as improving swimming technique, LLMs have shown potential superior to that of standard models [26]. This suggests LLMs may be more adept at knowledge delivery than at driving complex physiological adaptations.

### The AI Safety Paradox: Why LLMs Can Be Both Dangerous and Overly Conservative

Safety is the cornerstone of exercise prescription [43]. The most alarming finding of this review is the consistent evidence from multiple independent *in silico* studies of potential systemic safety flaws in LLMs. These studies reveal that LLMs can generate dangerous advice that directly violates clinical contraindications [27–29] or recommends unsafe training parameters. The root cause is not merely informational error but a fundamental architectural flaw: LLMs are probabilistic “imitators,” not “clinical reasoners” capable of causal inference [44]. They lack the ability to conduct a nuanced risk-benefit analysis for an individual’s unique pathophysiological state, particularly during pre-participation health screening [43]. These safety failures can be categorized as errors of knowledge (the model is unaware of a contraindication) and errors of reasoning (the model knows the risk factors but fails to apply this knowledge logically when generating a plan).

This leads to a troubling duality: on one side, reckless recommendations that could cause harm, and on the other, a tendency to “sacrifice training effectiveness for the sake of excessive safety,” [9] as noted in one large-scale expert evaluation. This oscillation between being directly dangerous and overly conservative exposes a profound absence of clinical judgment, which may stem from the inherent tension between a model’s generative capability and its alignment strategies aimed at being “harmless” [45].

Beyond these planning-level risks, our analysis uncovers a largely overlooked yet more fundamental safety deficit: current LLMs are purely text-based models, utterly incapable of assessing, guiding, or correcting the biomechanical quality of an exercise. A plan that appears perfect on paper can lead directly to injury [46] if core movements like a squat or deadlift are performed with improper form. This chasm between the “textual plan” and its “physical execution” is a massive and insidious Achilles’ heel. It implies that even if LLMs could perfectly generate textual plans, they could never independently ensure the fundamental safety of the exercise process as long as they cannot perceive and provide feedback on the user’s physical movements. We strongly urge that future research must pivot toward multimodal AI (e.g., incorporating computer vision for posture assessment) to bridge this critical text-physical divide.

### Beyond Ienstructions: Can LLMs Become Our ‘Behavior Change Partners’?

Despite limitations in their core prescriptive tasks, the HCI studies included in this review reveal the immense potential of LLMs in another dimension: as ‘behavior change partners’ that empower users and foster motivation. Whether through narrating data to enhance self-reflection [33] or co-creating plans via dialogue [32], these studies point to a common mechanism: they support users’ psychological needs for autonomy and competence, rather than treating them as passive recipients of instructions.

This can be understood through established theoretical lenses. According to Self-Determination Theory (SDT), supporting these needs is key to igniting intrinsic motivation and long-term adherence [46]. Meanwhile, the Technology Acceptance Model (TAM) [47] reminds us that adoption is not solely dependent on objective quality; user trust and individual decision-making styles are critical moderating variables [11]. These findings suggest a paradigm shift for AI health systems, from simple “information delivery” to “intrinsic motivation empowerment”, where the core task is to systematically enhance a user’s capability, opportunity, and motivation to drive sustainable behavior change.

### Ethical, Legal, and Health Equity Implications

The application of LLMs in this high-stakes domain raises profound ethical, legal, and health equity challenges that warrant specific attention. Key issues include navigating technological paternalism versus user autonomy, establishing clear lines of liability when harm occurs, and addressing the risk that these technologies may exacerbate existing health inequalities through the digital divide and algorithmic bias [48, 49]. Without careful governance, the promise of “democratizing” coaching may fail, widening the gap between the health-advantaged and the underserved. A detailed analysis of these challenges, framed by principles of bioethics and health equity, is provided in Appendix F.

### Strengths and Limitations of this Review

To our knowledge, this is the first systematic review to comprehensively evaluate the efficacy, quality, and safety of LLMs for personalized exercise recommendation. Its strengths include a timely synthesis of recent literature, an interdisciplinary perspective bridging citation silo between computer science and sports medicine, and a rigorous risk of bias assessment for all included studies.

However, the review is constrained by the limitations of the primary studies, many of which had small sample sizes, short durations, or high risk of bias. The rapid pace of LLM development means our findings are a snapshot in time. The marked heterogeneity of the included studies necessitated a narrative synthesis, which carries an inherent risk of subjectivity, though we employed a structured framework to mitigate this. Finally, like all reviews, this work may be subject to publication bias, and our search strategy, despite its breadth, may have missed relevant studies, particularly from non-English sources or specialized databases not included in our search.

### Implications for Future Research and Practice

Based on our findings, we propose the following implications. For researchers, there is an urgent need for more rigorous, long-term RCTs, the development of standardized evaluation frameworks, and a pivot towards multimodal AI to address safety. For practitioners, we advocate for a human-AI synergistic paradigm, guided by the risk stratification framework presented in Box 1, where LLMs serve as assistive tools under strict professional oversight. This synergistic paradigm is strongly supported by recent evidence from Puce et al. [50], who evaluated ChatGPT-4 generated training plans for elite swimmers. Their study revealed a critical nuance: while AI performed well in designing low-intensity aerobic sessions, it struggled with the complex periodization required for high-intensity zones (e.g., anaerobic threshold and power). Crucially, 65% of coaches found the plans usable only with modifications. This underscores a key implication for practice: AI tools should be deployed to handle standardized, data-heavy tasks (like drafting basic aerobic cycles), thereby freeing up human coaches to focus on complex, contextsensitive decision-making (like high-intensity load management and real-time adaptation). For developers, we recommend a strategic shift from pursuing the “optimal prescription” to creating a “behavior change partner,” focusing on user engagement and implementing robust safety guardrails. Ultimately, professional bodies must develop position stands to guide the responsible integration of this powerful but imperfect technology into practice.

Box 1 Az User Risk Stratification Framework for the Clinical Application of LLMs in Exercise Prescription

This framework provides clinicians with a practical, risk-based guide for applying Large Language Models (LLMs) in the context of exercise prescription. It is adapted from the core logic of the ACSM’s preparticipation health screening guidelines.

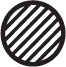
 Green Light Zone: Low-Risk Individuals

– **Applies to:** Asymptomatic, apparently healthy adults with no known underlying cardiovascular, metabolic, or renal disease **and no history of exercise-induced symptoms (e.g., chest pain, dizziness)**.
– **Permitted Use:** As a tool for general fitness information, exercise idea generation, and creating preliminary, non-specific workout templates.
– **Key Requirement:** User self-responsibility. All suggestions should be critically evaluated by the user based on common sense and general fitness knowledge. The LLM acts as an “inspiration tool,” not a definitive authority.
– **Example Scenario:** *A healthy 30-year-old looking for a 5k running schedule*.

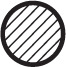
 Amber Light Zone: Moderate-Risk Individuals

– **Applies to:** Individuals with **controlled** chronic conditions (e.g., **stable hypertension stage 1, type 2 diabetes with HbA1c < 7%**), older adults (**> 65 years**) with mild functional limitations, or those with a history of musculoskeletal injury.
– **Permitted Use: Strictly prohibited for independent use.** An LLM’s output may serve as a preliminary draft or a starting point for discussion between the patient and a qualified professional.
– **Key Requirement: Mandatory expert oversight.** Any plan or suggestion generated by an LLM **must** be thoroughly reviewed, modified, and explicitly approved by a qualified health or exercise professional (e.g., physician, physical therapist, clinical exercise physiologist) before implementation.
– **Example Scenario:** *A 55-year-old with controlled hypertension seeking low-impact cardio options*.

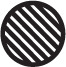
 Red Light Zone: High-Risk Individuals

– **Applies to:** Individuals with unstable or complex comorbidities (e.g., **recent myocardial infarction < 3 months, uncontrolled diabetes, symptomatic heart failure**), or any absolute contraindications to exercise (e.g., acute pulmonary embolus, retinal detachment risk).
– **Permitted Use: Completely prohibited.** The use of general-purpose LLMs for any form of exercise guidance should be strictly avoided.
– **Key Requirement: Exclusive reliance on human experts.** All exercise prescriptions must originate directly from the patient’s dedicated medical or rehabilitation team.
– **Example Scenario:** *A patient with recent myocardial infarction or proliferative diabetic retinopathy*.

## CONCLUSIONS

This systematic review, through a comprehensive assessment of the preliminary yet rapidly evolving body of empirical research, reveals a fundamental paradox surrounding the application of large language models (LLMs) to personalized exercise recommendation—the “AI Prescription Paradox.” On one hand, LLMs show immense potential for empowering novel user interactions and democratizing access to health knowledge. On the other, our findings clearly indicate that LLMs exhibit concerning potential deficits across three core dimensions: their efficacy in driving long-term physiological adaptation is as yet insufficient and often inferior to that of human experts; their output quality suffers from a pervasive lack of comprehensiveness; and most alarmingly, they possess significant and even dangerous vulnerabilities in clinical safety.

We therefore conclude definitively that LLMs, in their present state, cannot and should not be considered autonomous or safe replacements for the core decision-making and supervisory roles of human exercise and health professionals. Based on this cautious, evidence-based judgment, we call for a fundamental paradigm shift across academia, industry, and clinical practice: a move away from the unrealistic pursuit of “full AI automation” and toward the construction of a human-centered, AI-augmented “human-AI synergistic paradigm” [35].

## Supplementary Material

The AI recommendation paradox: a systematic review evaluating the promise, peril, and path forward for large language models in exercise recommendation

## References

[1] World Health Organization. Physical Activity and Sedentary Behaviour: A Brief to Support People Living with Type 2 Diabetes. Geneva: World Health Organization; 2022.

[2] Pedersen BK, Saltin B. Exercise as medicine – evidence for prescribing exercise as therapy in 26 different chronic diseases. Scand J Med Sci Sports. 2015; 25(suppl 3):1–72. doi: 10.1111/sms.12581. 26606383

[3] American College of Sports Medicine. Guidelines for Exercise Testing and Prescription. 4^th^ ed. Philadelphia, PA: Lea & Febiger; 1991.

[4] Cerin E, Leslie E. How socio-economic status contributes to participation in leisure-time physical activity. Soc Sci Med. 2008; 66(12):2596–2609. doi: 10.1016/j.socscimed.2008.02.012. 18359137

[5] Vaswani A, Shazeer N, Parmar N, et al. Attention is all you need. In: Advances in Neural Information Processing Systems. Vol 30. Curran Associates, Inc.; 2017:6000–6010.

[6] Lee P, Bubeck S, Petro J. Benefits, limits, and risks of GPT-4 as an AI chatbot for medicine. N Engl J Med. 2023; 388(13):1233–1235. doi: 10.1056/NEJMc2304040. 36988602

[7] Topol E. Deep Medicine: How Artificial Intelligence Can Make Healthcare Human Again. 1^st^ ed. New York, NY: Basic Books; 2019.

[8] Zaleski A, Berkowsky RS, Craig K, Pescatello L. Comprehensiveness, accuracy, and readability of exercise recommendations provided by an AI-based chatbot: mixed methods study. JMIR Med Educ. 2024; 10(1):e51308. doi: 10.2196/51308. 38206661 PMC10811574

[9] Dergaa I, Ben Saad H, El Omri A, et al. Using artificial intelligence for exercise prescription in personalised health promotion: a critical evaluation of OpenAI’s GPT-4 model. Biol Sport. 2024; 41(2):221–241. doi: 10.5114/biolsport.2024.133661. 38524814 PMC10955739

[10] Nutbeam D, Lloyd JE. Understanding and responding to health literacy as a social determinant of health. Annu Rev Public Health. 2021; 42:159–173. doi: 10.1146/annurev-publhealth-090419-102529. 33035427

[11] van Arum S, Genç HU. Selective trust: Understanding human-AI partnerships in personal health decision-making process. Int J Hum Comput Stud. 2025; 181:103152. doi: 10.1016/j.ijhcs.2024.103152.

[12] Düking P, Sperlich B, Voigt L, et al. ChatGPT generated training plans for runners are not rated optimal by coaching experts, but increase in quality with additional input information. J Sports Sci Med. 2024; 23(1):56–72. doi: 10.52082/jssm.2024.56. 38455449 PMC10915606

[13] Havers T, Masur L, Isenmann E, et al. Reproducibility and quality of hypertrophy-related training plans generated by GPT-4 and Google Gemini as evaluated by coaching experts. Biol Sport. 2025; 42(2):289–329. doi: 10.5114/biolsport.2025.145911. 40182716 PMC11963122

[14] Rahimi SA, Cwintal M, Huang Y, et al. Application of artificial intelligence in shared decision making: scoping review. JMIR Med Inform. 2022; 10(8):e36199. doi: 10.2196/36199. 35943793 PMC9399841

[15] An R, Shen J, Wang J, Yang Y. A scoping review of methodologies for applying artificial intelligence to physical activity interventions. J Sport Health Sci. 2024; 13(3):428–441. doi: 10.1016/j.jshs.2023.09.010. 37777066 PMC11116969

[16] Lai X, Chen J, Lai Y, et al. Using large language models to enhance exercise recommendations and physical activity in clinical and healthy populations: scoping review. J Med Internet Res. 2025; 27:e59309. doi: 10.2196/59309. PMC12133071 40424584

[17] Puce L, Bragazzi NL, Currà A, Trompetto C. Harnessing generative artificial intelligence for exercise and training prescription: applications and implications in sports and physical activity—a systematic literature review. Appl Sci. 2025; 15(7):3497. doi: 10.3390/app15073497.

[18] Page MJ, McKenzie JE, Bossuyt PM, et al. The PRISMA 2020 statement: an updated guideline for reporting systematic reviews. BMJ. 2021; 372:n71. doi: 10.1136/bmj.n71. 33782057 PMC8005924

[19] Popay J, Roberts H, Sowden A, et al. Guidance on the Conduct of Narrative Synthesis in Systematic Reviews: A Product from the ESRC Methods Programme Version 1. Lancaster: Lancaster University; 2006. doi: 10.13140/2.1.1018.4643.

[20] Braun V, Clarke V. One size fits all? What counts as quality practice in (reflexive) thematic analysis? Qual Res Psychol. 2021; 18(3):328–352. doi: 10.1080/14780887.2020.1769238.

[21] Pajo LS, Rabuya R, Andacao A, Tuano AMS, Lobo J. A 10-week large language model-generated (LLM) versus human-made volleyball training program on the jumping performance of collegiate volleyball athletes. J Phys Educ. 2025; 36:e3611. doi: 10.4025/jphyseduc.v36i1.3611.

[22] Genç A, Aydin R, Canuzakov K, et al. Digital coaches: an alternative to expert coaches for men’s fitness goals. Phys Act Rev. 2025; 13(2):35–44. doi: 10.16926/par.2025.13.18.

[23] Masagca RC. The AI coach: a 5-week AI-generated calisthenics training program on health-related physical fitness components of untrained collegiate students. J Hum Sport Exerc. 2025; 20(1):39–56. doi: 10.55860/13v7e679.

[24] Ebrahimi E, Rashidy P, Mohammadalinezhad SE, Hajizadeh R. The effect of a 6-week AI-generated core stability training program on balance and flatfoot in blind female students. J Asian Paralympic Movement. 2024; 4(2):83–91.

[25] Philuek P, Kusump S, Sathianpoonsook T, et al. The effects of Chat GPT generated exercise program in healthy overweight young adults: a pilot study. J Hum Sport Exerc. 2024; 20(1):169–179. doi: 10.55860/1epqgp77.

[26] Comendant C. Large Language Model-Based Sport Coaching System Using Retrieval-Augmented Generation and User Models [Bachelor’s thesis]. Enschede, Netherlands: University of Twente; 2024.

[27] Akrimi S, Schwensfeier L, Düking P, Kreutz T, Brinkmann C. ChatGPT-4o-generated exercise plans for patients with type 2 diabetes mellitus—assessment of their safety and other quality criteria by coaching experts. Sports. 2025; 13(4):92. doi: 10.3390/sports13040092. PMC12031090 40278718

[28] Xu Y, Liu Q, Pang J, et al. Assessment of personalized exercise prescriptions issued by ChatGPT 4.0 and intelligent health promotion systems for patients with hypertension comorbidities based on the transtheoretical model: a comparative analysis. J Multidiscip Healthc. 2024; 17:5063–5078. doi: 10.2147/JMDH.S477452. 39539514 PMC11559245

[29] Saraç H, Ulusoy İT, Alpay J, Ödemiş H, Söğüt M. Evaluating the potential role of AI chatbots in designing personalized exercise programs for weight management. Int J Hum Comput Interact. Published online February 27, 2025:1–8. doi: 10.1080/10447318.2025.2462752.

[30] Erol E, Arıkan H. Does ChatGPT provide comprehensive and accurate information regarding the effects, types and programming of core exercises? Turk J Kinesiol. 2024; 10(3):178–182. doi: 10.31459/turkjkin.1516614.

[31] Wachholz F, Manno S, Schlachter D, Gamper N, Schnitzer M. Acceptance and trust in AI-generated exercise plans among recreational athletes and quality evaluation by experienced coaches: a pilot study. BMC Res Notes. 2025; 18(1):112. doi: 10.1186/s13104-025-07172-9. 40082991 PMC11908068

[32] Shin D, Hsieh G, Kim YH. PlanFitting: personalized exercise planning with large language model-driven conversational agent. In: Proceedings of the 7th ACM Conference on Conversational User Interfaces. ACM; 2025:1–19. doi: 10.1145/3719160.3736607.

[33] Strömel KR, Henry S, Johansson T, Niess J, Woźniak PW. Narrating fitness: Leveraging large language models for reflective fitness tracker data interpretation. In: Proceedings of the CHI Conference on Human Factors in Computing Systems. ACM; 2024:1–16. doi: 10.1145/3613904.3642032.

[34] Haag D, Kumar D, Gruber S, et al. The last JITAI? Exploring large language models for issuing just-in-time adaptive interventions: Fostering physical activity in a prospective cardiac rehabilitation setting. In: Proceedings of the 2025 CHI Conference on Human Factors in Computing Systems. ACM; 2025:1–18. doi: 10.1145/3706598.3713307.

[35] Shneiderman B. Human-Centered AI. 1^st^ ed. Oxford: Oxford University Press; 2022.

[36] Wang D, Churchill E, Maes P, et al. From human-human collaboration to human-AI collaboration: designing AI systems that can work together with people. In: Extended Abstracts of the 2020 CHI Conference on Human Factors in Computing Systems. ACM; 2020:1–6. doi: 10.1145/3334480.3381069.

[37] Puce L, Ceylan Hİ, Trompetto C, et al. Optimizing athletic performance through advanced nutrition strategies: Can AI and digital platforms have a role in ultraendurance sports? Biol Sport. 2024; 41(4):305–313. doi: 10.5114/biolsport.2024.141063. 39416500 PMC11475005

[38] Wackerhage H, Schoenfeld BJ, Hamilton DL, Lehti M, Hulmi JJ. Stimuli and sensors that initiate skeletal muscle hypertrophy following resistance exercise. J Appl Physiol. 2019; 126(1):30–43. doi: 10.1152/japplphysiol.00685.2018. 30335577

[39] Zourdos MC, Klemp A, Dolan C, et al. Novel resistance training–specific rating of perceived exertion scale measuring repetitions in reserve. J Strength Cond Res. 2016; 30(1):267–275. doi: 10.1519/JSC.0000000000001049. 26049792

[40] Stone M, Plisk S, Collins D. Strength and conditioning: training principles: evaluation of modes and methods of resistance training – a coaching perspective. Sports Biomech. 2002; 1(1):79–103. doi: 10.1080/14763140208522788. 14658137

[41] Roberts BM, Nuckols G, Krieger JW. Sex differences in resistance training: a systematic review and meta-analysis. J Strength Cond Res. 2020; 34(5):1448–1460. doi: 10.1519/JSC.0000000000003521. 32218059

[42] Gao Y, Xiong Y, Gao X, et al. Retrieval-augmented generation for large language models: a survey. arXiv. Preprint posted online March 27, 2024. doi: 10.48550/arXiv.2312.10997.

[43] Riebe D, Franklin BA, Thompson PD, et al. Updating ACSM’s recommendations for exercise preparticipation health screening. Med Sci Sports Exerc. 2015; 47(11):2473–2479. doi: 10.1249/MSS.0000000000000664. 26473759

[44] Grote T, Berens P. On the ethics of algorithmic decision-making in healthcare. J Med Ethics. 2020; 46(3):205–211. doi: 10.1136/medethics-2019-105586. 31748206 PMC7042960

[45] Huang L, Yu W, Ma W, et al. A survey on hallucination in large language models: principles, taxonomy, challenges, and open questions. ACM Trans Inf Syst. 2025; 43(2):1–55. doi: 10.1145/3703155.

[46] Ryan GA, Snarr RL, Eisenman ML, Rossi SJ. Seasonal training load quantification and comparison in college male soccer players. J Strength Cond Res. 2022; 36(4):1038–1045. doi: 10.1519/JSC.0000000000003589. 32304515

[47] Davis FD. Perceived usefulness, perceived ease of use, and user acceptance of information technology. MIS Q. 1989; 13(3):319–340. doi: 10.2307/249008.

[48] Obermeyer Z, Powers B, Vogeli C, Mullainathan S. Dissecting racial bias in an algorithm used to manage the health of populations. Science. 2019; 366(6464):447–453. doi: 10.1126/science.aax2342. 31649194

[49] Price WN, Cohen IG. Privacy in the age of medical big data. Nat Med. 2019; 25(1):37–43. doi: 10.1038/s41591-018-0272-7. 30617331 PMC6376961

[50] Puce L, Żmijewski P, Cotellessa F, et al. The role of artificial intelligence in sports training: opportunities, challenges and future applications for competitive swimming. Biol Sport. 2026; 43(1):355–367. doi: 10.5114/biolsport.2026.152352. 41668944 PMC12884889

