# Supplementary material for: The AI recommendation paradox: a systematic review evaluating the promise, peril, and path forward for large language models in exercise recommendation
Source: Biol Sport. 2026 Mar 4;43:949–61. doi: 10.5114/biolsport.2026.158676 (PMC13343266; doi:10.5114/biolsport.2026.158676)
Supplement: The AI recommendation paradox: a systematic review evaluating the promise, peril, and path forward for large language models in exercise recommendation [file JBS-43-57447-s1.pdf]

SUPPLEMENTARY

Appendix A: Detailed Systematic Search Strategy and Results
Search Period: From database inception until June 18, 2025.

No.	Database / Source	Final Search Query String / Method	Search Results (Hits)
1	PubMed	(“Large Language Model” [tiab] OR LLM [tiab] OR LLMs [tiab] OR “Generative AI” [tiab] OR “Generative Artificial Intelligence” [tiab] OR “Generative Pre-trained Transformer” [tiab] OR ChatGPT [tiab] OR “GPT-3” [tiab] OR “GPT-4” [tiab] OR Gemini [tiab] OR Bard [tiab] OR Claude [tiab] OR LLaMA [tiab] OR Mistral [tiab] OR Grok [tiab] OR Qwen [tiab] OR Deepseek [tiab] OR “Conversational AI” [tiab] OR Chatbot [tiab] OR “Virtual Coach” [tiab] OR “AI Coach” [tiab]) AND (“Exercise Prescription” [tiab] OR “Training Plan” [tiab] OR “Training Program” [tiab] OR “Fitness Plan” [tiab] OR “Exercise Program” [tiab] OR “Exercise Regimen” [tiab] OR “Workout Plan” [tiab] OR “Workout Program” [tiab] OR “Hypertrophy Program” [tiab] OR “Hypertrophy Plan” [tiab] OR “Strength Program” [tiab] OR “Strength Plan” [tiab])	34
2	Web of Science (WoS) Core Collection	TS=(“Large Language Model” OR LLM OR LLMs OR “Generative AI” OR “Generative Artificial Intelligence” OR “Generative Pre-trained Transformer” OR ChatGPT OR “GPT-3” OR “GPT-4” OR Gemini OR Bard OR Claude OR LLaMA OR Mistral OR Grok OR Qwen OR Deepseek OR “Conversational AI” OR Chatbot OR “Virtual Coach” OR “AI Coach”) AND TS=(“Exercise Prescription” OR “Training Plan” OR “Training Program” OR “Fitness Plan” OR “Exercise Program” OR “Exercise Regimen” OR “Workout Plan” OR “Workout Program” OR “Hypertrophy Program” OR “Hypertrophy Plan” OR “Strength Program” OR “Strength Plan”)	48
3	Scopus	TITLE-ABS-KEY(“Large Language Model*” OR llm OR llms OR “Generative AI” OR “Generative Artificial Intelligence” OR “Generative Pre-trained Transformer*” OR chatgpt OR “GPT-3” OR “GPT-4” OR gemini OR bard OR claude OR llama OR mistral OR grok OR qwen OR deepseek OR “Conversational AI” OR chatbot* OR “Virtual Coach*” OR “AI Coach*”) AND TITLE-ABS-KEY(“Exercise Prescription” OR “Training Plan*” OR “Training Program*” OR “Fitness Plan*” OR “Exercise Program*” OR “Exercise Regimen*” OR “Workout Plan*” OR “Workout Program*” OR “Hypertrophy Program*” OR “Hypertrophy Plan*” OR “Strength Program*” OR “Strength Plan*”)	263
4	IEEE Xplore	(“Large Language Model” OR LLM OR LLMs OR “Generative AI” OR “Generative Artificial Intelligence” OR “Generative Pre-trained Transformer” OR ChatGPT OR “GPT-3” OR “GPT-4” OR Gemini OR Bard OR Claude OR LLaMA OR Mistral OR Grok OR Qwen OR Deepseek OR “Conversational AI” OR Chatbot OR “Virtual Coach” OR “AI Coach”) AND (“Exercise Prescription” OR “Training Plan” OR “Training Program” OR “Fitness Plan” OR “Exercise Program” OR “Exercise Regimen” OR “Workout Plan” OR “Workout Program” OR “Hypertrophy Program” OR “Strength Program”)	42
5	ProQuest Dissertations & Theses Global	abstract(“Large Language Model” OR LLM OR LLMs OR “Generative AI” OR “Generative Artificial Intelligence” OR “Generative Pre-trained Transformer” OR ChatGPT OR “GPT-3” OR “GPT-4” OR Gemini OR Bard OR Claude OR LLaMA OR Mistral OR Grok OR Qwen OR Deepseek OR “Conversational AI” OR Chatbot OR “Virtual Coach” OR “AI Coach”) AND abstract(“Exercise Prescription” OR “Training Plan” OR “Training Program” OR “Fitness Plan” OR “Exercise Program” OR “Exercise Regimen” OR “Workout Plan” OR “Workout Program” OR “Hypertrophy Program” OR “Hypertrophy Plan” OR “Strength Program” OR “Strength Plan”)	20
6	medRxiv & bioRxiv	A systematic search of 11 validated simple-paired queries was performed in the Abstract or Title field with the “all” operator (e.g., ChatGPT “Training Program”). The combined search yielded 55 initial hits.	55 (Combined)
7	SportRxiv	A broad search was conducted using a validated Concept A string: (“Large Language Model” OR LLM OR LLMs OR “Generative AI” OR “Artificial Intelligence” OR ChatGPT OR “GPT-4” OR Gemini OR Chatbot OR “Virtual Coach” OR “AI Coach”)	10

No.	Database / Source	Final Search Query String / Method	Search Results (Hits)
8	arXiv.org	A two-step search was conducted in the advanced search "all fields" category.<br>1. High-precision search using Concept A and Concept B terms yielded 0 results.<br>2. High-sensitivity search using Concept A and broader exercise terms (e.g., Exercise, Fitness) yielded 0 results.	0
9	Clinical Trial Registries (ClinicalTrials.gov & WHO ICTRP)	An iteratively refined, high-precision query was used: ("Large Language Model" OR LLM OR ChatGPT OR "Generative AI" OR "Virtual Coach" OR Chatbot) AND ("Exercise Prescription" OR "Training Plan" OR "Training Program" OR "Physical Activity Plan" OR "Workout Plan" OR "Fitness Plan")	105
	**TOTAL RAW HITS (Before Deduplication)**		**577**
	**RECORDS AFTER DEDUPLICATION**		**294**

Legend for Search Concepts:
1. Concept A (AI Terms): Refers to the set of keywords related to Large Language Models and associated AI technologies as listed in the query strings (e.g., "Large Language Model", ChatGPT, "Generative AI", etc.).
2. Concept B (Exercise Terms): Refers to the set of keywords related to exercise prescription and training plans as listed in the query strings (e.g., "Exercise Prescription", "Training Plan", "Workout Program", etc.).

Appendix B: Custom Quality Assessment Checklist for Non-Interventional Study Designs

Introduction and Rationale

To ensure a comprehensive and rigorous quality assessment across the diverse range of studies included in this review, we developed a customized checklist for study designs not amenable to standard tools like the Cochrane RoB 2 or ROBINS-I. This includes *in silico* expert evaluation studies, Human-Computer Interaction (HCI) and usability studies, and qualitative research. The development of this checklist was guided by established principles from relevant fields, including the AXIS tool for cross-sectional studies, established heuristics for HCI and usability evaluation [1], and the foundational trustworthiness criteria for qualitative research proposed by Lincoln and Guba [2]. The checklist is divided into three sections tailored to the specific methodological considerations of each study type.

Scoring and Application

For each study, two independent reviewers completed the relevant checklist section. A final quality rating was assigned based on a holistic assessment of the responses. This was not a quantitative scoring system, but a qualitative judgment of the study's overall methodological rigor and risk of bias.

- **High Quality / Low Risk of Bias:** The study satisfactorily addressed most or all of the relevant domains. Its methods were transparent and appropriate, and its conclusions were well-supported by the data presented.
- **Moderate Quality / Moderate Risk of Bias:** The study provided some information on most domains but had notable shortcomings in one or more key areas (e.g., lack of blinding, poor description of the sample, low inter-rater reliability) that could potentially bias the results.
- **Low Quality / High Risk of Bias:** The study had significant flaws in multiple domains or failed to report critical methodological details, seriously undermining the confidence in its findings.

B1: Checklist for *In Silico* Expert Evaluation Studies

Assessment Domain	Guiding Questions and Considerations for Reviewers
1. **Clarity and Complexity of User Persona(s)**	• Was the hypothetical user/patient persona clearly and adequately described?<br>• Did the persona's complexity (e.g., presence of comorbidities, specific goals, contraindications) match the study's research question and allow for a meaningful evaluation of the LLM's capabilities?
2. **Transparency and Reproducibility of Prompting Strategy**	• Was the full, verbatim prompt(s) provided?<br>• Was the prompting strategy (e.g., single-shot, multi-turn conversational, use of specific personas in the prompt) clearly described and justified?<br>• Was the process repeatable by other researchers?
3. **Qualifications and Blinding of Expert Evaluators**	• Were the qualifications, expertise, and number of evaluators clearly stated and appropriate for the evaluation task?<br>• Was the evaluation process blinded? (i.e., Did the evaluators know the origin of the plans they were assessing – AI vs. human, GPT-4 vs. Gemini, etc.?)
4. **Validity and Reliability of Evaluation Instrument**	• Was the tool or rubric used for evaluation clearly described?<br>• Was it based on established guidelines (e.g., ACSM, NSCA), a validated instrument, or developed specifically for the study?<br>• Was inter-rater reliability (e.g., Kappa, ICC) assessed and reported? If so, was it adequate?
5. **Overall Methodological Rigor**	• Did the study design include a meaningful comparator (e.g., human expert, other AI, established guidelines)?<br>• Was the analysis of the evaluation data appropriate and clearly reported?

B2: Checklist for Human-Computer Interaction (HCI) and Usability Studies

Assessment Domain	Guiding Questions and Considerations for Reviewers
1. **Clarity of HCI Research Question / Objective**	• Was the goal of the study clearly defined (e.g., to assess usability, user experience, trust, acceptance)?
2. **Appropriateness of Participant Sampling and Characteristics**	• Were the participants representative of the target user group for the technology?<br>• Were the recruitment method, sample size, and participant demographics clearly described?
3. **Realism and Clarity of the User Task / Procedure**	• Was the task that users performed with the system clearly described and ecologically valid (i.e., representative of real-world use)?<br>• Was the study context (e.g., lab, field) described?
4. **Appropriateness and Rigor of Data Collection Methods**	• Were the data collection methods (e.g., think-aloud protocol, validated surveys like SUS/UMUX, interviews, system logs) appropriate for the research question?<br>• Were validated instruments used where applicable?
5. **Systematic Nature of Data Analysis**	• Was the method of data analysis (e.g., thematic analysis for qualitative data, appropriate statistical tests for quantitative data) clearly described and systematically applied?

B3: Checklist for Qualitative Research (e.g., Interviews, Focus Groups)

This section is based on the Lincoln and Guba (1985) trustworthiness framework.

Trustworthiness Criterion	Guiding Questions and Considerations for Reviewers
1. **Credibility (Confidence in the "Truth" of the Findings)**	• Were methods used to enhance credibility described? (e.g., prolonged engagement with participants, peer debriefing, member checking, triangulation of data sources).
2. **Transferability (Applicability to Other Contexts)**	• Was a "thick description" of the participants and the research context provided, allowing readers to judge the applicability of the findings to their own settings?
3. **Dependability (Consistency and Repeatability of the Process)**	• Was the research process described in sufficient detail to allow for an "audit trail"? (i.e., Could another researcher follow the process, from data collection to analysis?).
4. **Confirmability (Objectivity; Findings Stem from Data, Not Bias)**	• Were steps taken to ensure that the findings are grounded in the data?<br>• Did the authors acknowledge their own potential biases or preconceptions (i.e., a statement of reflexivity)?

References for Appendix B

[1] Nielsen, J. (1994). *Usability Engineering*. Morgan Kaufmann.
[2] Lincoln, Y. S., & Guba, E. G. (1985). *Naturalistic Inquiry*. Sage Publications.

Appendix C: Summary of Risk of Bias and Quality Assessment for Included Studies

This table presents the overall methodological quality rating for each included study, based on the appropriate assessment tool.

Study ID (Author, Year)	Study Design	Assessment Tool	Overall Risk of Bias / Quality Rating	Key Rationale for Rating
		**RCTs / Experiments**		
Genç et al. (2025)	RCT	Cochrane RoB 2	**High Risk of Bias**	Critical risk due to major systematic differences in intervention delivery (supervision vs. unsupervised).
Philuek et al. (2025)	RCT (Pilot Study)	Cochrane RoB 2	**High Risk of Bias**	Critical risk from extremely small sample size (N=9) and high risk across multiple RoB domains.
Strömel et al. (2024)	Online Experiment (RCT)	Cochrane RoB 2	**Low Risk of Bias**	Well-designed, large online RCT with validated measures and transparent reporting.
		**Quasi-experimental**		
Comendant (2024)	Quasi-experimental (pre-post)	ROBINS-I	**Serious Risk of Bias**	Critical risk from lack of baseline comparability control and small sample size (N=10).
Ebrahimi et al. (2025)	Quasi-experimental	ROBINS-I	**Serious Risk of Bias**	Critical risk as AI effects are inseparable from significant human assistance; "Scopus AI" unclear.
Masagca (2025)	Quasi-experimental	ROBINS-I	**Serious Risk of Bias**	Critical risk from non-randomization and a complete lack of baseline fitness control.
Pajo et al. (2025)	Quasi-experimental	ROBINS-I	**Moderate Risk of Bias**	Non-randomization; uncontrolled baseline confounding; no adherence monitoring.
		**In Silico / Expert Eval.**		
Akrimi et al. (2025)	In silico expert eval.	Custom Checklist	**Low Quality**	Critical risk due to extremely small evaluator sample (N=3, authors) and no blinding.
Dergaa et al. (2024)	In silico expert eval.	Custom Checklist	**Moderate Quality**	Unparalleled expert panel size, but main risk from non-blinded, author-evaluators.
Düking et al. (2023)	In silico expert eval.	Custom Checklist	**Moderate Quality**	Strong design, but main risk from low reported inter-rater reliability among experts.
Erol & Arıkan (2024)	In silico expert eval.	Custom Checklist	**Moderate Quality**	Assessed general knowledge, not prescription; risk from lack of inter-rater reliability reporting.
Haag et al. (2025)	In silico eval.	Custom Checklist	**High Quality**	Landmark study; rigorous design with blinding, personas, and advanced statistical analysis.
Havers et al. (2025)	In silico expert eval.	Custom Checklist	**Moderate Quality**	Innovative design, but risk from lack of evaluator blinding and low inter-rater reliability.
Oliveira (2024)	In silico expert eval.	Custom Checklist	**High Quality**	Strong methodology with real-world scenarios, blinding, and instrument validation.
Saraç et al. (2025)	In silico expert eval.	Custom Checklist	**Low Quality**	Main strength is objective parameter comparison; serious risk from very small expert group (N=3).
Washif et al. (2024)	In silico expert eval.	Custom Checklist	**Moderate Quality**	Author-evaluators and no blinding; clear prompt engineering focus.
Xu et al. (2024)	In silico expert eval.	Custom Checklist	**High Quality**	Excellent methodology with real-world scenarios and a large, multidisciplinary expert panel.
Zaleski et al. (2024)	In silico expert eval.	Custom Checklist	**High Quality**	Rigorous instrument based on gold-standard (ACSM); quantified key quality gaps.
		**HCI / Qualitative**		
Jones et al. (2024)	System Design & Pilot Study	Custom Checklist	**High Quality (Design Contribution)**	Novel system design; empirical findings have low evidence strength due to N=2 pilot.
Larbi et al. (2024)	Qualitative (Interviews + Focus Groups)	Custom Checklist	**High Quality**	Rigorous qualitative analysis; valuable user needs for obese population.
Larbi et al. (2025)	Usability Testing	Custom Checklist	**Low Quality**	Critical risk from very small (N=5), non-target user sample and lack of analysis detail.

Study ID (Author, Year)	Study Design	Assessment Tool	Overall Risk of Bias / Quality Rating	Key Rationale for Rating
Shin et al. (2025)	System Design & Multi-faceted Eval.	Custom Checklist	High Quality (User Study)	User study (N=18) is high quality; expert evaluation portion (N=3) is low quality.
van Arum et al. (2025)	Mixed-methods (Survey + Interviews)	Custom Checklist	High Quality	Very strong methodology with validated scales and large N for both quant/qual components.
Wachholz et al. (2025)	Mixed-methods (Survey + Interviews)	Custom Checklist	Moderate Quality	Survey has risk from convenience sample; interview findings have low evidence strength (N=6).

Legend: Low Risk of Bias / High Quality; Moderate Risk of Bias / Moderate Quality; High/Serious Risk of Bias / Low Quality

Appendix D: Risk of bias assessment for included Randomized Controlled Trials (RCTs) using the Cochrane RoB 2 tool. FIG appendix

(A) Weighted summary plot of risk-of-bias judgments for each domain. The bar chart summarizes the proportion of studies assessed as having low risk, some concerns, or high risk of bias for each of the five domains and for the overall risk of bias.
(B) Traffic light plot of domain-level risk-of-bias judgments for each included RCT. Each row represents an individual study, and each column represents a bias domain. Green, yellow, and red indicate low risk, some concerns, and high risk of bias, respectively.

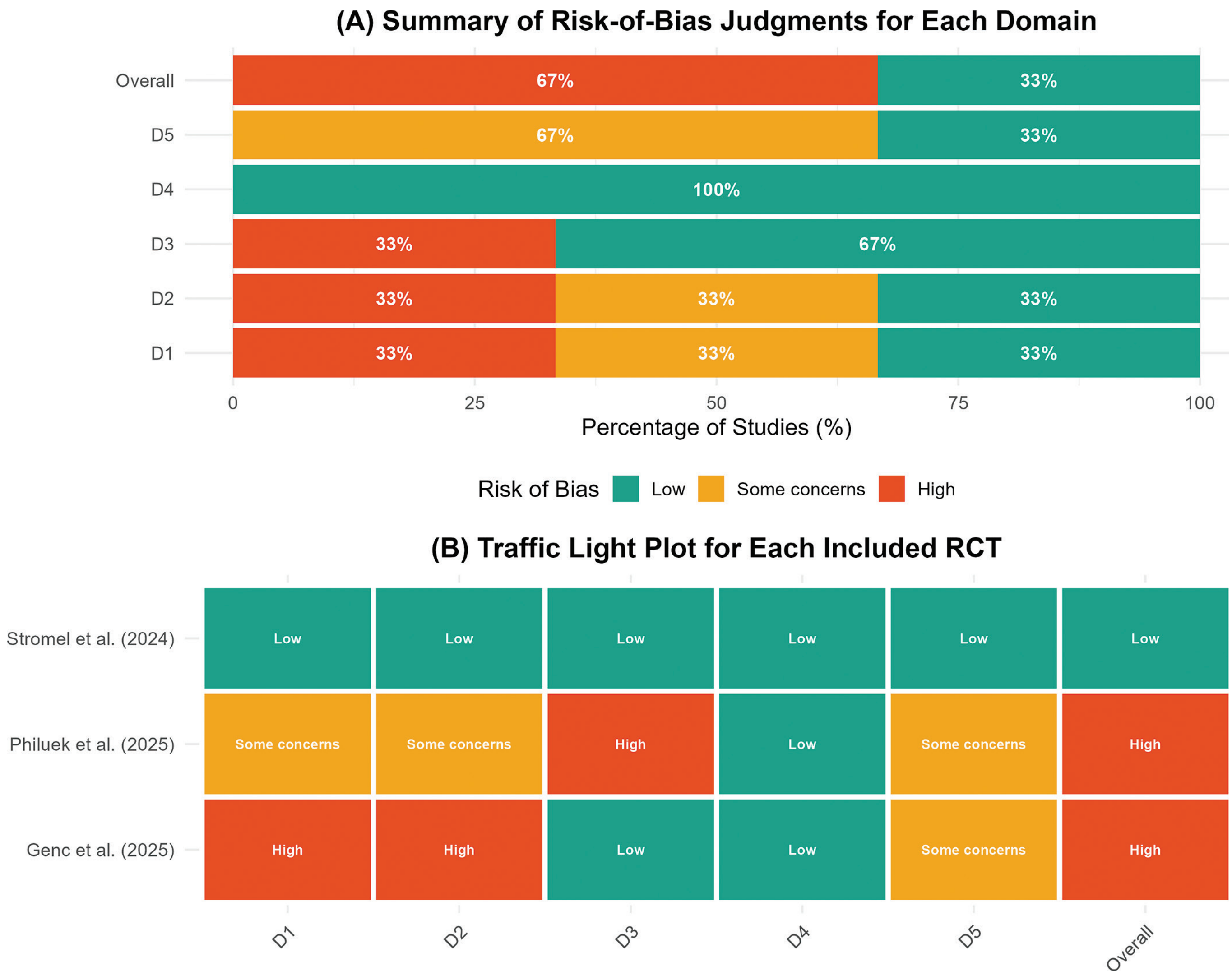

Appendix E: Box 2 – From a Flawed AI Draft to a Clinically Sound Plan: A Case Example of Human-AI Synergy

This box illustrates the practical application of the Human-AI Synergy paradigm. It shows how a qualified professional can transform a potentially unsafe, generic AI-generated plan into a safe, effective, and personalized recommendation for a moderate-risk individual.

Patient Profile (Amber Light Zone ◍):

- **Name:** John, 58 years old.
- **Goal:** Improve cardiovascular health and general strength.
- **Condition:** Medically managed, stable hypertension (Blood Pressure ~135/85 mmHg with medication). Sedentary lifestyle. No other known diseases.
- **User Prompt to LLM:** "Give me a 3-day per week beginner workout plan to get healthier."

Part 1: Initial AI-Generated Draft (ChatGPT-4)

Day	Activity	Sets & Reps / Duration	Notes
Day 1	**Cardio & Upper Body**		
	Treadmill Run	30 minutes at 70–80% Max HR	Push yourself to a challenging pace.
	Bench Press	3 sets of 8–12 reps	
	Overhead Press	3 sets of 8–12 reps	Keep your core tight.
	Pull-ups	3 sets to failure	
Day 2	**Rest**		
Day 3	**Cardio & Lower Body**		
	HIIT on Bike	20 min (1 min on, 1 min off)	Go all out during the 'on' intervals.
	Barbell Squats	3 sets of 8–12 reps	Go deep for full range of motion.
	Deadlifts	3 sets of 6–8 reps	Lift heavy.
Day 4	**Rest**		
Day 5	**Full Body**		
	Burpees	5 sets of 10 reps	Explosive and fast.
	Kettlebell Swings	3 sets of 15 reps	
	Plank	3 sets, hold as long as possible	
